# Supplementary material for: Causal Relationship between Aging and Anorexia Nervosa: A White-Matter-Microstructure-Mediated Mendelian Randomization Analysis
Source: Biomedicines. 2024 Aug 16;12(8):1874. doi: 10.3390/biomedicines12081874 (PMC11351342; doi:10.3390/biomedicines12081874)
Supplement: Supplementary file 1 [file biomedicines-12-01874-s001.zip › Supplementary Figure/Figure S8/Leaveoneout plot of MR analysis for TL to ubm-b-1907.pdf]

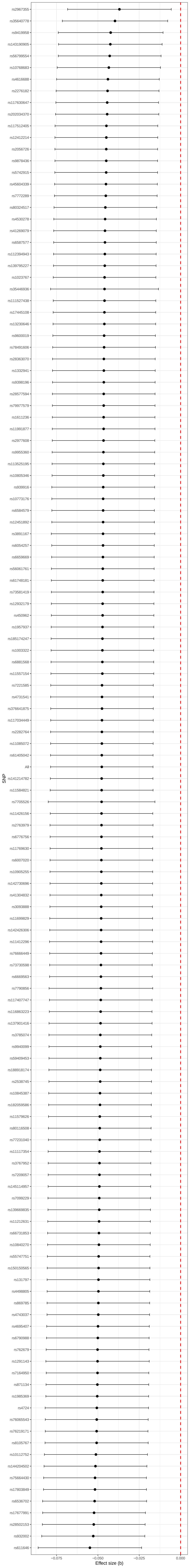
rs2967355
rs35640778
rs9419958
rs143190905
rs56799554
rs10768683
rs4616688
rs2276182
rs117630647
rs202034370
rs117512405
rs12412214
rs2056726
rs9878436
rs5742915
rs45604339
rs7772289
rs80324517
rs4530278
rs41269079
rs6587577
rs112394943
rs139795227
rs1023767
rs35446936
rs111527438
rs17445108
rs13230666
rs9600019
rs78491606
rs28363070
rs1332941
rs9398196
rs28577594
rs79977579
rs1611236
rs11991877
rs2977608
rs9955360
rs113525195
rs10805346
rs939916
rs10773176
rs6584579
rs12451892
rs3891167
rs6054257
rs6659669
rs56061761
rs61748181
rs73581419
rs12932179
rs450962
rs1957937
rs185174247
rs1003322
rs6881568
rs11557154
rs7221585
rs4731541
rs376641875
rs117034449
rs2282764
rs11085072
rs61405042
All
rs141214782
rs11584821
rs7705526
rs11426156
rs2763979
rs6776756
rs11769630
rs6007020
rs10905255
rs142730696
rs41304832
rs3093888
rs11699829
rs142426306
rs11412296
rs76666449
rs73730598
rs6669563
rs7790856
rs117407747
rs116863223
rs137901416
rs3785074
rs9940099
rs59409453
rs188918174
rs2538745
rs10845387
rs182059586
rs11579626
rs80116508
rs77231040
rs11117354
rs3767952
rs7209057
rs145114957
rs7099229
rs139669835
rs11212631
rs66731853
rs10840270
rs55747751
rs150150565
rs131797
rs4498805
rs869785
rs4743037
rs4695407
rs6790988
rs762679
rs1291143
rs7164950
rs871134
rs1985369
rs4724
rs76065543
rs76219171
rs8105767
rs10112752
rs144204502
rs75664430
rs17803849
rs6536702
rs17677991
rs28502153
rs932002
rs611646
SNP
−0.075
−0.050
−0.025
0.000
Effect size (b)
